# The miR-20-Rest-Wnt signaling axis regulates neural progenitor cell differentiation

**DOI:** 10.1038/srep23300

**Published:** 2016-03-21

**Authors:** Yi Cui, Jin Han, Zhifeng Xiao, Tong Chen, Bin Wang, Bing Chen, Sumei Liu, Sufang Han, Yongxiang Fang, Jianshu Wei, Xiujie Wang, Xu Ma, Jianwu Dai

**Affiliations:** 1 Reproductive and Genetic Center of National Research Institute for Family Planning, Beijing 100081, China; 2 State key Laboratory of Molecular Developmental Biology, Institute of Genetics and Developmental Biology, Chinese Academy of Sciences, Beijing 100190, China; 3 University of Chinese Academy of Sciences, Beijing 100049, China; 4 The State Key Laboratory of Plant Genomics, Institute of Genetics and Developmental Biology, Chinese Academy of Sciences, Beijing 100101, China; 5 State Key Laboratory of Veterinary Etiological Biology, Key Laboratory of Veterinary Public Health of Ministry of Agriculture, Lanzhou Veterinary Research Institute, CAAS, Lanzhou 730046, China

## Abstract

Increasing evidence suggests that three dimensional (3-D) cell cultures are an improvement over traditional two dimensional (2-D) cell cultures. Current researches have extensively focused on the study of utilizing biomaterial-based 3-D culture systems to study and direct stem-cell fate both *in vitro* and *in vivo*. Here in our study, we screened the differential expression patterns of miRNAs between 2-D cultured and 3-D cultured NPCs using microarray analysis. Among these differentially expressed miRNAs, miR-20 was found to increase during differentiation of NPCs. Specifically, the facilitative effect on neural differentiation of miR-20 is mediated, at least in part by directly target the Rest gene, which is essential for preventing neural differentiation and maintaining NPCs self-renewal. Furthermore, the expression of miR-20 was decreased when the WNT pathway was inhibited by knock down of β-catenin or by exogenous Dkk protein, whereas it increased when the WNT pathway was activated by exogenous Wnt3a protein. Overall, miR-20, Rest and Wnt signaling are suggested to be involved in a regulatory circuit that can modulate the neural differention of NPCs. This novel regulatory circuit provides additional insight into how microRNAs interact with signaling molecules during neural differentiation of NPCs, allowing for fine-tuning of intricate cellular processes.

Considerable attention has focused on the study of neural progenitor cells (NPCs) because of their potential as a renewable cell source for clinical nervous tissue repair^1^. Various experiments have demonstrated that the properties of stem cells are precisely controlled by the stem cell niche^2^,^3^. Three-dimensional cell culture systems represent a reconstituted niche that can provide a precise spatiotemporal substrate that supports the cell growth, organization, and differentiation of NPCs either on or within their structure^4^,^5^. Most studies on NPCs have relied on analysis of cells grown in 2D cell-culture models that fail to reconstitute the *in vivo* cellular microenvironment. Our previous studies have shown that the collagen sponge scaffold has a good biocompatibility with NPCs and also the cell behavior of NPCs is markedly affected when cultured on the scaffold. When NPCs cultured in collagen sponge based 3-D system, it may yield higher clone formation efficiency and expressed less neuron marker Tuj1 than 2-D cultured NPCs in differentiation medium without growth factors^6^. Results from previous studies indicated that 3-D collagen sponge based system contributes to matintain the self-renewal properties of NPCs^6^,^7^. Unraveling the exact molecular mechanisms by which NPCs renew themselves in 3-D cultured systems will provide new insights into both basic neurosciences and the clinical applications of stem cell-based therapies for neurodegenerative diseases.

NPCs are capable of self-renewal and can give rise to both neurons and glia^8^,^9^. Growing evidence has demonstrated that miRNAs play a central role in controlling the balance between self-renewal and differentiation. MiRNAs are particularly abundant in the brain and are temporally expressed during neural differentiation^10^,^11^,^12^. Increasing evidence suggests that miRNA gene expression can be changed as a response to the microenvironment of the cell. Our analyses have shown that the miRNA expression patterns differ extensively between traditional 2-D culture systems and 3-D culture systems. MiRNAs are small non-coding RNAs that influence diverse biological functions through the repression of target genes^13^,^14^. To identify the exact molecular mechanisms by which these miRNAs regulate cell function, we constructed an miRNA-gene network using the TargetScan algorithm^15^. The miRNA-gene network analysis indicated that the RE1-silencing transcription factor (Rest) gene was regulated by miR-20. By gain-of-function and loss-of function approaches, we showed that the endogenous levels of Rest are negatively controlled by miR-20 in NPCs. REST is a repressor of neuronal genes during embryonic development and is known to block neural differentiation by binding to and inhibiting the expression of neuronal genes. Previous studies have demonstrated that silencing Rest *in vitro* enhances the rate of differentiation and subsequent maturation of NPCs^16^,^17^.

Considering the previous report revealing that the repression of Wnt and Wnt receptor genes is an important candidate mechanism by which REST maintains the pluripotent state^18^, we hypothesized that Wnt signaling may be involved in attenuating the neural differentiation of 3-D cultured NPCs. The Wnt signaling network is known to regulate many cellular processes and to play essential roles in NPCs. In our study, we observed that activation of canonical Wnt signaling by exogenous Wnt3a may reverse the inhibitory effect on neural differentiation by miR-20. By contrast, DKK1, a negative regulator of Wnt signaling, may reverse the effect that miR-20 mimics had on promoting neural differentiation. To our knowledge, there is no reported relationship between miR-20 and Wnt signaling. The quantitative real-time PCR data in this study showed that miR-20 expression is elevated when Wnt signaling is activated by Wnt3a, whereas miR-20 expression is reduced when Wnt signaling is inhibited by knock down of β-catenin or by exogenous DKK-1, a specific antagonist of the Wnt/β-catenin pathway.

In summary, we showed that miR-20 inhibited the differentiation of NPCs by negatively targeting the transcriptional repressor gene *Rest*. Our findings further suggested that Wnt signaling is involved in maintaining the self-renewal capacity of 3-D cultured NPCs. An understanding of the molecular mechanisms underlying the self-renewal and differentiation of 3-D cultured NPCs is helpful for applying the NPCs in future clinical uses *in vivo*.

## Results

### Good cellular biocompatibility of collagen sponge scaffolds

**Figure 1:**
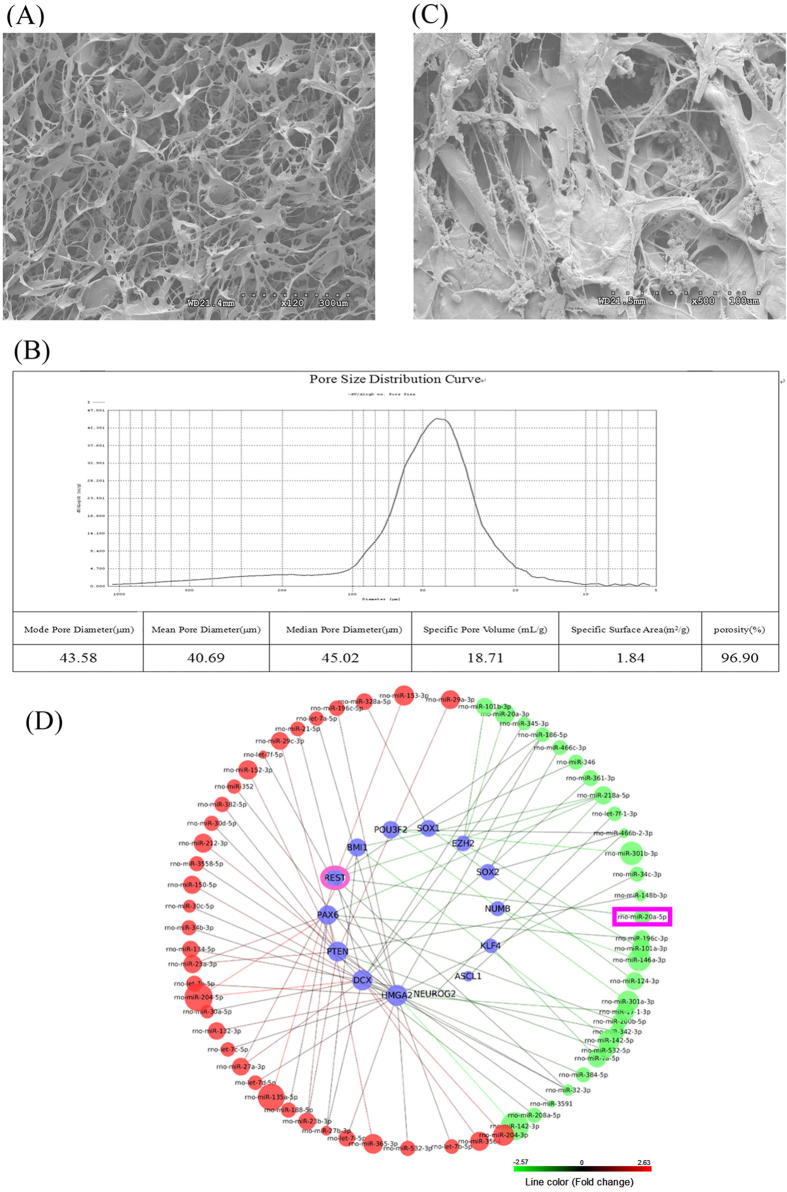
The morphology and characteristic of collagen sponge scaffold and 3-D cultured NPCs. (**A**) Scanning electron microscopy (SEM) images of the collagen sponge scaffold. (**B**) Evaluation of pore size distribution and porosimetry by mercury porosimetry. (**C**) The morphology of the 3-D cultured NPCs was observed by SEM. (**D**) MiRNA array profiles of differentially regulated miRNAs and their target genes, which were identified in NPCs seeded on 2-D and 3-D substrates using bioinformatic analysis. The node sizes and line colors are correlated with expression changes of the miRNAs. MiRNAs covered by red nodes were up-regulated in 3D cultured NPCs and miRNAs covered by green nodes were down-regulated in 3D cultured NPCs.

The mercury porosimetry is widely used to determine various quantifiable aspects of the material’s nature, such as pore diameter, pore volume, surface area, and porosity^19^,^20^. Also, the scanning electron microscopy (SEM) is capable of observing the microstructure of biomaterial. By combining two techniques of analysis our results indicated that the collagen sponge scaffolds had an irregular multi-porous structure defined by smooth surface collagen walls with a mean pore size of 40.69 μm in diameter, thus providing a suitable substrate for cell attachment (Fig. 1A,B). The large pores provided an environment in which the cells were not too tight and had good spreading, and multiple layers of cells could grow in multiple directions. NPCs could be grown in the pores or could adhere to the surface of the collagen sponge with good biocompatibility (Fig. 1C). Our study showed that the collagen sponge scaffold provided a superior substrate for the attachment and growth of NPCs. The scaffold enabled cells to grow at various angles and thus allowed for multiple directions of movement. These observations were different from those for 2D cultured cells, where the cells were too confluent and grew as a monolayer.

### High-throughput miRNA profiling in 2D and 3D cultured NPCs

To better understand whether miRNAs are involved in the self-renewal of stem cells and the fate determination of NPCs in 3-D culture systems, we used a miRNA microarray to identify the differential miRNA expression patterns of NPCs in 2-D cultures and 3-D cultures. By comparing the miRNA profiles, 179 miRNAs were found to have differential expressions in 2-D and 3-D cultured NPCs. These results indicated that the altered expression of a substantial number of miRNAs could affect the expression of a large number of genes related to NPC self-renewal. Using the miRNA-target gene pairs predicted by TargetScan, we constructed the regulatory network between 30 DE (differential expression) miRNAs and the fourteen key genes related to self-renewal (Fig. 1D). The altered expression of miRNAs may explain the regulatory mechanism of self-renewal and differentiation in 3-D cultured NPCs. Among these differentially regulated miRNAs, we found that miR-20 was down-regulated in 3-D cultured NPCs, which was consistent with previous results that showed the down-regulation of miR-20 in 3-D cultured PA-1 cells^7^. Bioinformatic analyses indicated that the self-renewal related Rest gene was the downstream target of miR-20 and may contribute to the self-renewal properties of 3-D cultured NPCs.

### MiR-20 directly targets Rest in NPCs

**Figure 2:**
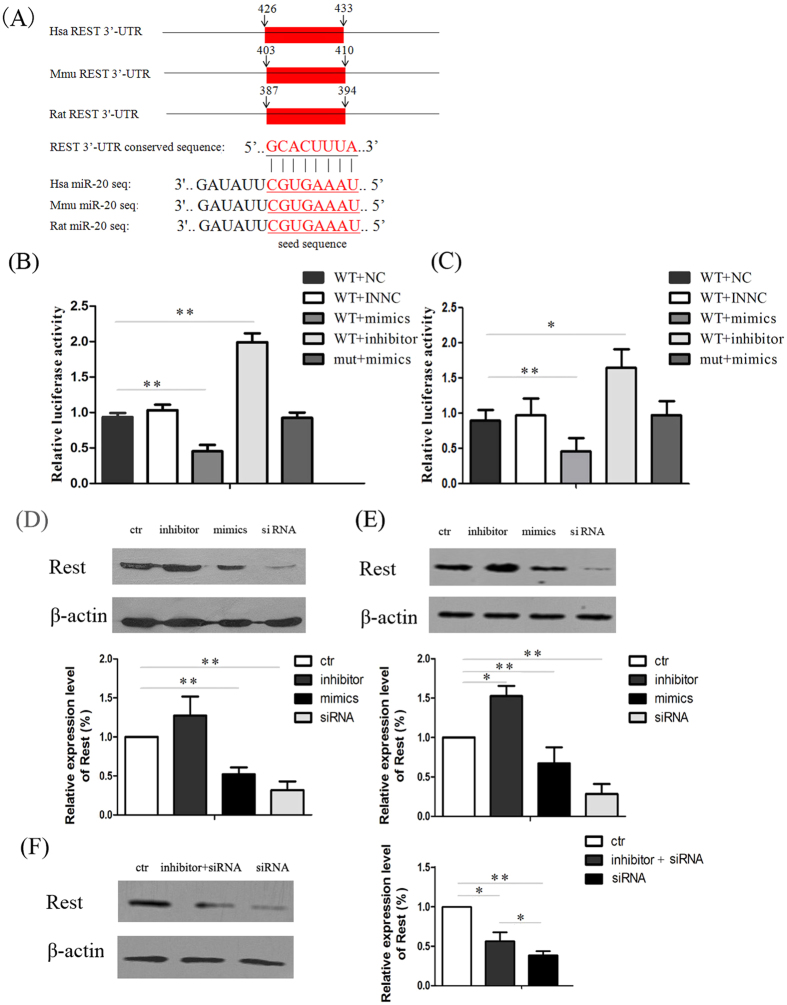
MiR-20 modulates Rest expression. (**A**) A schematic representation of putative miRNA-binding sites (shown in red) in the 3′UTR sequence of Rest. Sequence alignment indicated that miR-20 and its predicted binding site in the Rest 3′UTR are 100% conserved in vertebrates. The seed region is underlined. Results of the dual luciferase reporter assay using HeLa cells (**B**) and NPCs (**C**). Luciferase activity of Rest wild-type 3′UTR vectors (wt) or its mutant derivative lacking the miRNA binding sites (mut) in HeLa cells. The results were normalized with the pRL-CMV-Renilla Luciferase control. Relative luciferase level = (S luc/S renilla)/(C luc/C renilla). Luc, raw firefly luciferase activity; Renilla, internal transfection control renilla activity; S, sample; C, WT + NC group. The data are shown as the means ± SD. From 3 independent repetitions. *P < 0.05 versus WT + NC and **P < 0.01 versus the corresponding WT + NC. The western blot analysis showed that MiR-20 negatively regulated Rest protein expression in HeLa cells (**D**) and NPCs (**E**). (ctr: Control vector transfection; Inhibitor: miR-20 inhibitor; Mimics: miR-20 mimics; SiRNA: Rest siRNA;NC: negative control; INNC: inhibitor negative control) (**F**) Western blot assay indicated that the miR-20 inhibitor may rescue the inhibitory effect on the expression of Rest resulted by Rest siRNA. The datas are shown as the means ± SD. From 3 independent repetitions. *P < 0.05 versus ctr and **P < 0.01 versus ctr.

MiRNAs function by binding to the 3′UTRs of target mRNAs and often result in down regulation of protein translation. MiRNA-target prediction algorithms indicated miR-20 binding sites within the 3′UTR of the Rest gene. Significantly, the 3′UTR elements of Rest and the sequences of the miR-20 putative binding sites are extremely conserved among different species (mouse, rat, and human). By base-pairing complementation, we found that the 3′UTR of Rest encompasses the putative binding regions bearing significant complementarities against miR-20 (Fig. 2A). As shown in Fig. 2B,C, when NPCs and HeLa cells were cotransfected with Rest 3′-UTR containing mutated miR-20 binding sites and miRNA mimics (mut + mimics), the luciferase activity was increased by nearly 2-fold compared to cells cotransfected with wild type Rest 3′-UTR, which contained the miR-20 binding sites and miRNA mimics (wt + mimics). This study revealed significant down-regulation of Luc activity (40–60%) for Rest UTRs in HeLa cells (Fig. 2B) and NPCs (Fig. 2C) in the presence of ectopic miR-20. No down-regulation occurred when the miR binding sites in each of the potential target UTRs were mutated. Contrary to the decrease in UTR-LUC activity caused by miR-20 overexpression, miR-20 inhibitor increased LUC activity by approximately 1.5 ~ 2 fold. The results suggested that these binding sites are required for miRNA binding and activity. Next, we evaluated whether the modulation of miR-20 affected the levels of Rest protein. Western blot assay showed that transfection with the miR-20 mimics resulted in a decrease of Rest protein in both NPCs and HeLa cells. On the other hand, the transfection of miR-20 inhibitors resulted in increased levels of the Rest protein (Fig. 2D,E). Furthermore, we conducted rescue experiments by transfecting the miR-20 inhibitor and Rest siRNA simultaneously. The expression of Rest was a little elevated when NPCs were transfected with miR-20 inhibitor simultaneously compared to transfected with Rest siRNA alone, further supporting the notion that Rest is a direct target of miR-20 (Fig. 2F).

### The expression pattern of miR-20 and Rest during neural Differentiation in the 2-D and 3-D cultured systems

**Figure 3:**
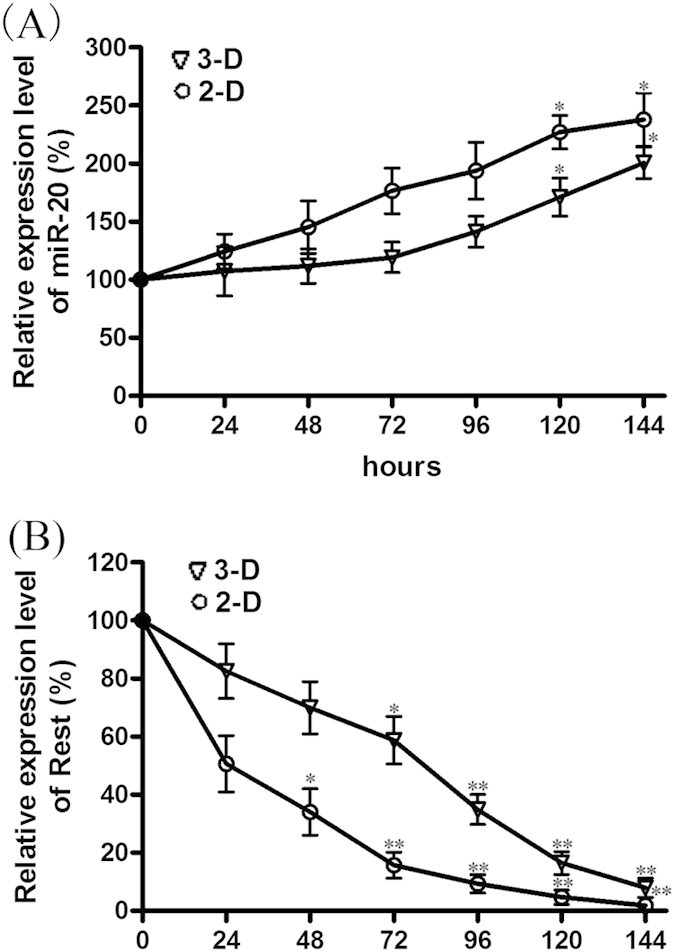
The expression pattern of miR-20 and Rest in 2-D and 3-D cultured NPCs. Quantitative real-time PCR analysis showed the time-dependent elevation of miR-20 mRNA levels during the differentiation process (**A**). In contrast, the mRNA levels of Rest were reduced in a time-dependent manner (**B**). The datas are shown as the means ± SD. From 3 independent repetitions. *P < 0.05 versus 0 and **P < 0.01 versus 0.

When evaluating the expression of both miR-20 and Rest, we found that the expression of miR-20 was elevated in a time-dependent manner during the differentiation process of NPCs both in the 2-D and 3-D culture systems, increasing by nearly 2-fold (p < 0.001) at day 6 when compared with undifferentiated cells (Fig. 3A). By contrast, the expression of the pluripotency factor REST markedly decreased in a time-dependent manner, which also corresponds with miR-20 up regulation (Fig. 3B). Notably, the expression of Rest was significantly down regulated at day 6 compared with undifferentiated cells. The expressions of miR-20 and Rest tended to vary less in 3-D cultured NPCs than in 2-D cultured cells, which helps to explain the inhibition of neural differentiation in the 3-D cultured NPCs.

### The Wnt signaling pathway is involved in the regulation of miR-20 in 3-D cultured NPCs

**Figure 4:**
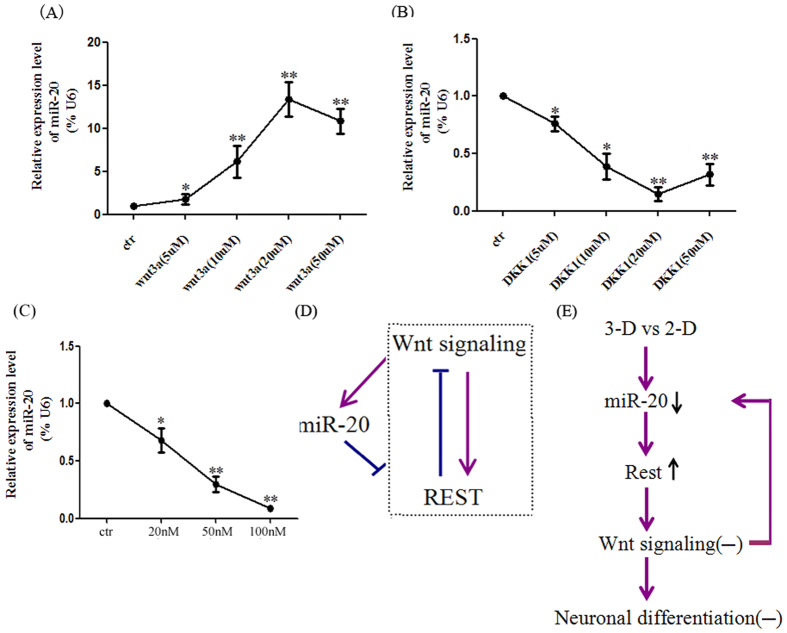
The regulatory circuit of miR-20, Rest and Wnt signaling. (**A**) Activation of Wnt signaling induced miR-20 activation. NPCs were treated with Wnt-3a or DKK1 and were harvested at the indicated times. Total RNA was extracted and miR-20 expression was measured by qPCR. The results were normalized to U6 RNA as an internal control. (**B**) A proposed model for the regulatory loop between miR-20, Rest and Wnt signaling in NPCs. The arrows represent Wnt activation and the bars represent repression. (**C**) The expression level of miR-20 was significantly attenuated when β-catenin was knocked down by siRNA in NPCs in a dose-dependent manner. (**D**) A working model for the relationship between miR-20, Rest and Wnt signaling involved in the neuronal differentiation of 3-D cultured NPCs. The data represent the means ± S.D. (n = 3). *P < 0.05 versus ctr and **P < 0.01 versus ctr.

Recent report showed that Rest plays an important role in regulating Wnt signaling^18^, though the relationship between Wnt signaling and miR-20 remains unclear. To confirm that Wnt signaling participates in miR-20 mediated neural differentiation, we initially determined whether the modulation of Wnt signaling with an agonist or inhibitor affected the expression of miR-20. As expected, the Real-time PCR analysis indicated that miR-20 expression was elevated when NPCs were treated with Wnt3a (Fig. 4A). In contrast, the miR-20 expression was reduced when NPCs were treated with DKK-1 (Fig. 4B). To further examine the functional importance of Wnt signaling on miR-20 expression, we silenced β-catenin via siRNA. As shown in Fig. 4C, transfection of NPCs with β-catenin siRNA significantly attenuated the expression level of miR-20. Our data provide the first evidence of a direct connection between Wnt signaling and miR-20. Additionally, the regulatory relationship between miR-20 and Rest was also confirmed by Western blot. REST has been reported to be a target of canonical Wnt signaling and could be induced by the addition of purified Wnt-3a^21^,^22^,^23^. We built a regulatory loop model of miR-20, Rest, and Wnt signaling, indicating that miR-20 may target the Rest gene and then inhibit Wnt signaling and that the inactivation of Wnt signaling can also suppress the Rest and miR-20 genes (Fig. 4D). In 3-D culture environments, the synergistic effects of miR-20, Rest, and Wnt signaling may be disturbed: the down regulation of miR-20 promotes the expression of Rest and then inhibits Wnt signaling, which contributes to the maintenance of self-renewal capacities in 3-D cultured neural stem cells (Fig. 4E).

### MiR-20 promotes neural differentiation of NPCs through inactivation of Rest

**Figure 5:**
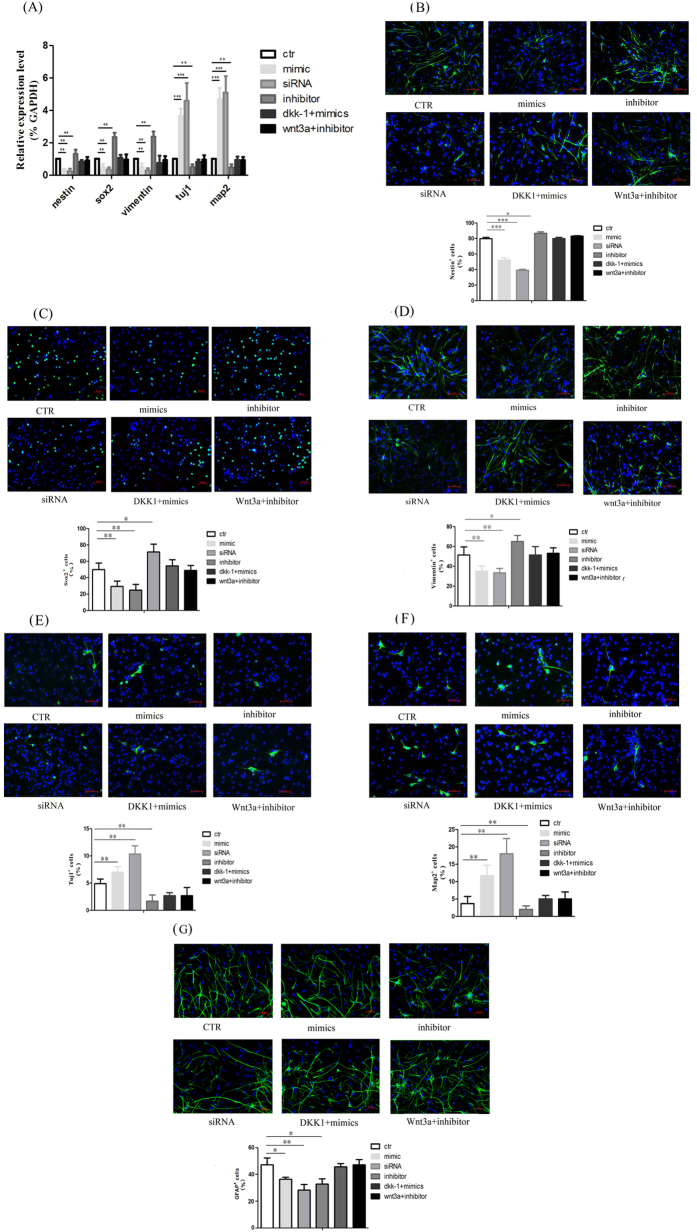
MiR-20 regulated NPCs differentiation. (**A**) qPCR data showing mRNA levels of Nestin, Sox2, Vimentin, Tuj1 and Map2 genes during NPCs differentiation. (**B–F**) Immunostaining images and quantified data of Nestin (**B**), Sox2 (**C**), Vimentin (**D**), Tuj1 (**E**) and Map2 (**F**) positive cells in NPCs transfected with miRNA mimics, miRNA inhibitor or Rest siRNA alone in differentiation medium or differentiation medium containing Wnt3a or DKK1 for 96 h. Scale bar, 50 μm (Top panel: immunostaining images; Bottom panel: quantified data from positive immunostaining cells). Quantitation and representative photomicrographs showed that *miR-20* promotes cell differentiation in NPCs. Bars show mean ± SD. All experiments were repeated three times. *P < 0.05 vs. ctr, **P < 0.01 vs. ctr, ***P < 0.001 vs. ctr.

**Figure 6:**
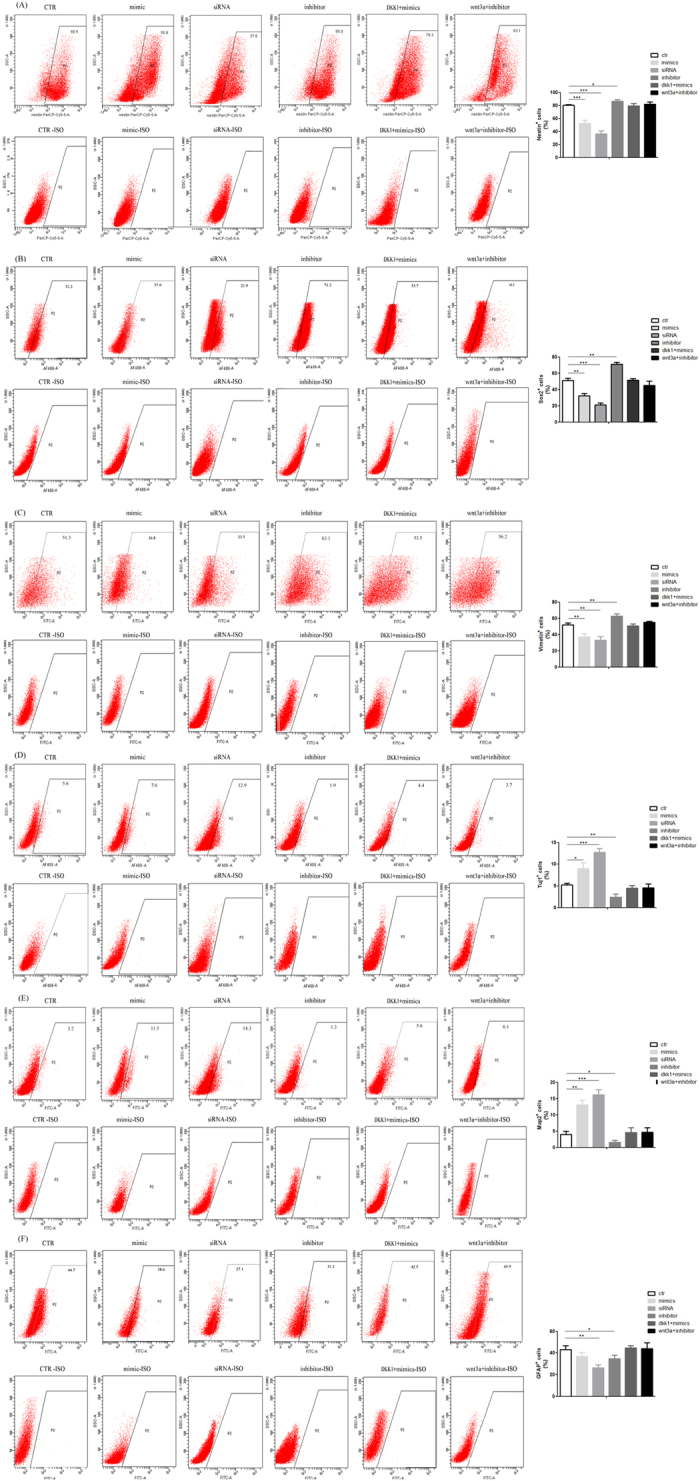
The percentage of Nestin, Sox2, Vimentin, Tuj1, Map2 and GFAP positive cells determined by Fluorescence-activated sorting (FACS) analysis. Representative images showed the expression level of these genes in NPCs transfected with miRNA mimics, miRNA inhibitor or Rest siRNA alone in differentiation medium or differentiation medium containing Wnt3a or DKK1 for 96 h. An isotype control is needed to determine whether fluorescence emitted is due to non-specific binding of the fluorescent antibody. The datas are shown as the means ± SD. From 3 independent repetitions. *P < 0.05 versus ctr, **P < 0.01 versus ctr, ***P < 0.001 vs. ctr.

To determine whether miR-20 influences neural differentiation, we explored the effect of miR-20 modulation on the percentage of Nestin+, Sox2+, Vimentin+, Tuj1+, Map2+ and GFAP+ cells via immunofluorescence staining in 2-D cultured NPCs. The fluorescence data revealed that the percentage of Nestin+, Sox2+ and Vimentin+ cells was increased by 10%, 21.7% and 13% in the miR-20 inhibitor group at 96 h after transfection compared to control group (p < 0.05) (Fig. 5B–D). Whereas, the percentage of Tuj1+ and Map2+ cells was significantly increased by 4% and 8% in the miR-20 mimics group compared to control group, respectively (p < 0.05) (Fig. 5E,F). Interestingly, the proportion of GFAP positive cell was not increased no matter whether miR-20 was over expressed or knocked down. It can be explanation that the over expressed miR-20 increases the population of mature neurons at the expense of GFAP-positive cells. Meanwhile when miR-20 was knocked down the differentiation of NPCs was inhibited and then the proportion of GFAP positive cell was decreaseed. The results of the flow cytometry analysis keep good agreement with the immunofluorescence staining results (Fig. 6). Next, we evaluated the role of Rest in the neural differentiation of NPCs. The efficiency of Rest silencing was confirmed by western blotting (Fig. 2D,E). Similar to the results for miR-20 overexpression, transfection with Rest siRNA also resulted in an increased percentage of Tuj1+ and Map2+ cells by 7% and 14%, respectively. Similar results were obtained when evaluating neural markers by quantitative real-time PCR during the differentiation of NPCs under various treatments (Fig. 5A).

It has been reported that Wnt3a and β-catenin play pivotal role in regulating the neural differentiation of NPCs^24^. Consistent with previous studies, our results showed that activation of Wnt/β-catenin signaling by exogenous Wnt3a promote neural differentiation of NPCs. In contrast, the neural differentiation was inhibited by knock down of β-catenin or exogenous DKK-1 (Fig. S1). Next we demonstrated that the effect of miR-20 in promoting neural differentiation could be antagonized by a negative regulator, DKK1, and the inhibitory effect of the miR-20 inhibitor on neural differentiation was antagonized by Wnt3a (Fig. 5).

### The role of miR-20 in 3-D cultured NPCs

**Figure 7:**
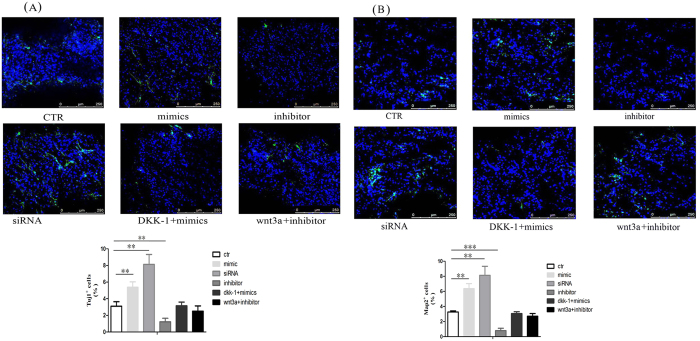
MiR-20 promoted neuronal differentiation in 3-D cultured NPCs. (**A,B**) Immunofluorescence detection of Tuj1 (**A**) and Map2 (**B**) positive cells in 3-D cultured NPCs after transfection with miR-20 mimics, inhibitor alone, or cultured in medium containing Wnt3a or DKK1. Scale bar, 250 μm (Left panel: immunostaining images; Right panel: quantified data from positive immunostaining cells). Bars show mean ± SD. All experiments were repeated three times. *P < 0.05 vs. ctr, **P < 0.01 vs. ctr, ***P < 0.001 vs. ctr.

To further explore the hypothesis that miR-20 participates in inhibiting the neural differentiation of 3-D cultured NPCs, we transfected miR-20 mimics, the miR-20 inhibitor, and Rest siRNA into 3-D cultured NPCs for 4 days. Consistent with previous results, the results of the immunofluorescence assay confirmed that the proportion of Tuj1+ and Map2+ cells increased in the miR-20 mimic group and the Rest siRNA group, whereas the proportion of these cells decreased in the miR-20 inhibitor group (Fig. 7). The effects that the miR-20 mimics and the miR-20 inhibitor had on promoting or inhibiting differentiation, respectively, could be compensated by culturing the transfected NPCs in differentiation medium containing Wnt3a or DKK1. These data not only support the previous observation that miR-20 plays an important role in neural differentiation but also demonstrate the regulatory relationship between miR-20 and Wnt signaling in 3-D cultured NPCs.

## Discussion

NSCs have become a research focus of many laboratories, but their biological characteristics and the mechanisms regulating their differentiation mechanisms are not fully clear. The microenvironment of NPCs can balance their quiescence with their self-renewal and proliferation, regulating their decision to differentiate. Though cells in tissues are organized into well-defined 3-D structures, most cell physiological studies are still performed on 2-D cell cultures that are far from the niche for the cells *in vivo*. Biomedical researchers have become increasingly aware of the limitations of conventional 2-dimensional tissue cell culture systems. Accordingly, 3-D culture system attracted increased attention because these systems enable cells to grow at various angles and thus allow for multiple directions of movement. Growing evidence has shown that the specific topologic architecture and geometry of a 3-D culture system influence cell phenotype and fate^25^,^26^. Our previous studies have demonstrated that the neural differentiation of NPCs was inhibited in comparison to NPCs cultured in conventional 2-D systems when NPCs were cultured in the collagen sponge scaffold prepared in our laboratory^25^,^26^.

Multiple studies have demonstrated that miRNAs have crucial roles in the self-renewal and differentiation of NPCs. The miRNA array profiling results indicated that the 3-D surface topography influencing the molecular behavior of NPCs may be mediated by miRNAs associated with maintaining stemness. Additionally, the 3-D architecture may regulate miRNAs involved in differentiation processes. The characterization of the miRNA pathways and their underlying molecular mechanisms is of great importance to understanding the effects of the 3-D collagen sponge system upon NPCs. One miRNA identified in the screen, miR-20, was of particular interest because it was down regulated in both PA-1 cells and NPCs in 3-D culture systems^7^. The results indicated that miR-20 is involved in regulating the ability of 3-D cultured cells to undergo neural differentiation, but the exact mechanisms of how miR-20 influences stem cell differentiation had been poorly understood. In our present work, we found that the expression of miR-20 was increased during neural differentiation. Previous studies have suggested that miR-20 is involved in the regulation of differentiation during embryonic development. The data of those studies clearly demonstrate that modulation of miR-20 expression, which is increased over the course of differentiation, can alter fate commitment during ES cell differentiation^27^.

Here, we provide compelling evidence that over-expression or knockdown of miR-20 alters neural differentiation by specifically regulating Rest protein levels. We combined computational and functional approaches to verify the specific effects of miR-20 on the regulation of the Rest gene. Earlier studies have demonstrated that Rest is essential for preventing precocious neural differentiation and maintaining NSC self-renewal in the adult hippocampus, and Rest was reported to be generally down-regulated during induction of neural differentiation^16^. The down regulation of REST generated more than twice the percentage of Tuj1 and Map2 positive cells compared to the controls^25^. Similar to the REST knockdown group, the results of our immunofluorescence assay revealed that a higher percentage of Tuj1 and Map2 positive cells were observed in the miR-20 mimic group compared to the control.

Various studies have implicated Wnt signaling in the control of cell growth and differentiation during central nervous system (CNS) development. Conditioned media containing active Wnt-3a proteins inhibit the regeneration of neurospheres but promote the differentiation of NPCs into Map2-positive neuronal cells^26^. Moreover, a blockade of Wnt signaling led to the inhibition of neural differentiation of cortical NPCs *in vitro* and in the developing mouse neocortex^28^. Numerous studies have demonstrated that Wnt signaling inhibits the self-renewal capacity of NPCs and instructively promotes their neural differentiation^29^,^30^. Nevertheless, the precise mechanism of how the Wnt pathway induces neural differentiation has not been elucidated until now. It has been reported that among the REST target genes, there are a large and significant number of genes encoding members of the Wnt signaling pathway^28^. To date, the degree to which REST is responsible for regulating the Wnt pathway remains unclear. Interestingly, REST expression can also be induced by the addition of purified Wnt-3a and Wnt-7a and can also be inhibited by the Wnt signaling antagonist Dickkopf. It seems likely that the regulatory relationship between Rest and the Wnt pathway is critical and can be mediated by many competing and reinforcing circuits that converge on this node in the transcriptional network. Using a Wnt inhibitor (dkk1) and agonist (Wnt3a), we established that Wnt signaling can successfully compensate for the effects of miR-20 on neural differentiation both in the 2-D and 3-D cultured systems. The real-time PCR results also provided the first evidence of a direct connection between the Wnt signaling pathway and miR-20. Taking into account the previous reports, we construct a complex circuit involving miR-20, REST, and Wnt signaling. MiR-20 negatively regulates the expression of Rest, which negatively regulates Wnt signaling in this complex circuit. On the other hand, Wnt signaling may positively regulate the expression of Rest and miR-20. This regulatory loop suggests that Rest promotes the ability of NPCs to self-renew either directly through inhibiting Wnt signaling or indirectly through downregulating the expression of miR-20. Fine control of the regulatory loop played an important role in maintaining an appropriate balance between self-renewal and differentiation in NPCs. The collagen sponge based 3-D culture system is not only a powerful tool for examining the effects of an external signal on NPCs but also has the potential to be an excellent and appealing resource for applications in regenerative medicine.

## Methods

### NPCS derivation, culture and differentiation

Rat NPCs were isolated from rat brain tissues according to a previous procedure with slight modifications^31^. NPCs were cultured in T25 flasks and suspended for growth in a growth medium consisting of Dulbecco’s modified Eagles medium (DMEM) plus Ham’s F-12 supplemented with 1% (v/v) antibiotic-antimycotic mixed stock solution, 2% (v/v) B-27 Supplement, 20 ng/mL EGF, 20 ng/mL bFGF at 37 °C in a humidified 5% (v/v) CO2 atmosphere. All animal experimental procedures were approved by the institutional review board of Institute of Genetics and Developmental Biology, Chinese Academy of Sciences, and performed in accordance with the Chinese Ministry of Public Health (CMPH) Guide for the care and use of laboratory animals. For 2-D cultures, the cells were seeded at a density of 3 × 10^5^ cells per well in poly-D-lysine coated 6-well plates. For 3-D culture, NPCs (1 × 10^6^) were added to a piece of collagen sponge scaffold. After 24 h adhesion, the adhesion medium was exchanged with differentiation medium (Dulbecco’s modified Eagles medium (DMEM) plus Ham’s F-12 supplemented with 1% (v/v) antibiotic-antimycotic mixed stock solution and 2% (v/v) B-27 Supplement). Cells were harvested for microarray and qPCR analyses after 4 days in culture.

Various different doses (5, 10, 20, and 50 μM) of Wnt3a or DKK1 (R&D Systems, Minneapolis, MN, USA) were added to the differentiation medium to evaluate their effects on the modulation of miR-20 expression or on the several properties of the NPCs. In each case, the range of concentrations was used as indicated.

### Collagen sponge scaffold preparation

The collagen sponge was made from bovine collagen of spongy bone tissue as described previously^32^. The collagen sponge was aseptically cut into pieces approximately 5 mm in diameter and 1 mm in thickness for cell culture. EDC (1-ethyl-3- (3-dimethylaminopropyl)-carbodiimide) cross-linking was performed for 4 h to increase the stability of the collagen sponge. The pore size distribution and porosimetry of coallgen sponge materials was evaluated by mercury porosimetry (PoreMasterGT 60, Quantachrome).

### Scanning electron microscopy

For scanning electron microscopy, the cells in the collagen sponge scaffold were fixed in 2% glutaraldehyde at 4°C overnight and prepared using conventional methods. After the critical drying point, the samples were sputter-coated with gold and evaluated under a scanning electron microscope (SEM) (S-3000N; Hitachi, Tokyo, Japan).

### miRNA microarray Analysis

The gene expression analysis was carried out using a μParaflo® miRNA microarray (MRA-1003, LC Sciences, Houston, TX, USA)^7^. The miRNA expression was quantified by subtracting the background noise of the raw data from the hybridization images and the data were normalized with LOWESS filtering (locally weighted regression)^33^. Fold-change ≥1.5 and p-value < 0.01 thresholds using Student’s t-test were used to sort out differentially expressed genes. MiRNA-target relationships were obtained from TargetScan (release 6.2), and the miRNA-gene network was constructed using Cytoscape (version 3.1.1). Node size and line color were correlated with the expression changes of miRNAs in NSCs cultured in the 2D and 3D systems. MiRNAs covered by red nodes were up-regulated in 3D cultured NPCs and miRNAs covered by green nodes were down-regulated in 3D cultured NPCs.

### DNA constructs and luciferase reporter assays

Luciferase assays were performed using standard approaches. To construct the miR-20 Luc reporter plasmid, a fragment of 3′-UTR of the Rest (1097 bp) gene containing the putative miR-20 binding site was cloned into a modified pGL3-promoter vector (Promega, Madison, WI, USA) which was modified according to previous reports^34^. The mutated 3′-UTR of Rest was generated using a site-directed mutagenesis kit (TransGen Biotech, Beijing, China). The full-length 3′-UTR and the mutated 3′-UTR of Rest was amplified by PCR using the primers listed in Table 1. All PCR products were digested at the SpeI and SphI sites before cloning into the pGL3-promoter vector. For transfection, HeLa cells and NPCs were seeded in 24-well plates in growth media and transfected using Lipofectamine 2000™ reagent. In each well, 0.5 μg of firefly luciferase vector, 0.03 μg of the Renilla luciferase (control vector), miR-20 mimics (10 nM) or miR-20 inhibitors (10 nM) were introduced. After 48 hours, firefly and Renilla luciferase activities were measured by dual-luciferase assays (Promega). All luciferase data are presented as the normalized ratio of luciferase/Renilla.

### RNA Extraction and Real-Time RT-PCR

Total RNA was isolated using TRIzol Reagent and the first-strand cDNA was synthesized using SuperScript™ III First-Strand Synthesis System (Invitrogen, Carlsbad, CA, USA). QPCR was performed using SYBR® Green PCR Master Mix (Roche, Mannheim, Germany) on a CFX96™ Real-Time PCR Detection System. Relative mRNA levels were determined and standardized with a GAPDH internal control using the 2^−ΔΔ^CT method^35^.

MiRNAs were extracted using the miRVana extraction kit (Ambion, Austin, TX, USA) and then reverse-transcribed and amplified using the microRNA reverse transcription and detection kit (Applied Biosystems, Inc. Foster City, CA). Primers for real-time PCR are all listed in Table 2. All results were normalized to U6 levels that were detected using the ABI miRNA U6 assay kit.

### Transfection of miRNA mimics, inhibitors, and small interfering RNAs

MiR-20 expression was modulated using the chemically synthesized miR-20 mimics or inhibitor modified by 2′-O-methyl (2′-O-Me) modifications (GenePharma Co., Ltd., Shanghai, China). The modified RNA oligonucleotides are resistant to a variety of ribo- and deoxyribonucleases in cultured cells, therefore the oligo-2′-O-Me-nucleotides form more stable hybrids with complementary RNA strands than equivalent RNA sequences^36^,^37^,^38^. Cells were transfected using 50 nM miRNA mimics, miRNA inhibitors, Negative Control, or Inhibitor Negative Control. NPC cells were transiently transfected with Rest or β-catenin siRNA (si-Rest: Invitrogen; si-β-catenin: Sigma Aldrich) or negative control siRNA (si-NC; Invitrogen and Sigma) according to the manufacturer’s instructions. In the RNAi experiments, 100 nM of Rest and β-catenin siRNA solution was transfected using X-tremeGENE siRNA Transfection Reagent.

### Western blot analysis

For western blot analysis, approximately 10 μg of proteins were loaded and separated on the BioRad mini gel system (Hercules, CA). The proteins were transferred to PVDF membranes. Protein expression of Rest and β-catenin was detected by incubating with antibodies anti-Rest (#07-579, Upstate), anti-beta-Actin (TA-09, ZSGB-BIO, Beijing, China) at a dilution of 1:1000 overnight at 4 °C in primary antibodies. The membranes were then incubated for 2 h at room temperature in HRP-labeled secondary antibody (PIERCE, Rockford, USA) (1:5000). The bands were visualized using colorimetric detection and exposure to autoradiography film.

### Immunofluorescence staining

The self-renewal and differentiation markers of the NPCs were also assessed by immunofluorescence^34^. For immunofluorescence staining analysis cells were incubated with the primary antibody *Nestin* (1:400; MAB353, Millipore), *Tuj1* (1:500,05-549, Upstate), Sox2 (1:100; 481400, Life technologies), Map2(1:400; M1406, Sigma), Vimentin (1:100; V6630, Sigma), GFAP (1:500; MAB360, millipore) overnight at 4 °C. The secondary antibodies are anti-mouse IgG FITC antibody (1:200, St. Louis, MO, USA) and anti-rabbit IgG FITC antibody (1:1000, St. Louis, MO, USA) diluted in blocking buffer. Nuclei are counter-stained with Hochest 33342 (1:500; 94403, St. Louis, MO, USA). The fluorescent images of 2-D cultured cells were visualized on a Zeiss 200 inverted fluorescent microscope (Carl Zeiss, Jena, Germany). The number of immunostained cells was counted in each of three random fields per well and the fluorescence images were selected randomly. The quantification of the immunofluorescence signal was performed by Image-Pro Plus software (Media Cybernetics, Bethesda, MD). The fluorescent images of 3-D cultured cells were taken with a Leica TCS SP5 scanning laser confocal fluorescence microscope (Leica Microsystems, Inc., Germany).

### Flow cytometry (FACS) analysis

The adherent NPC cells were digested it into single cell suspension and then fixed with BD Cytofix™ buffer (Cat. No. 554655) for 20 minutes at room temperature. The cells were permeabilized with BD Phosflow™ Perm Buffer I (Cat. No. 557885), and then stained with antibody Nestin (561231, Becton, Dickinson and Company;), Sox2 (ab75485, Cambridge, USA), Vimentin (ab128507, Cambridge, USA), Tuj1(ab195879, Cambridge, USA), Map2 (560399, Becton, Dickinson and Company), GFAP (ab4674, Cambridge, USA) and theire Isotype control (550795, Becton, Dickinson and Company; ab170190, Cambridge, USA; ab91356, Cambridge, USA; ab171464 Cambridge, USA; 557721, Becton, Dickinson and Company; ab37382, Cambridge, USA). Flow cytometry was performed on a BD LSR™ II flow cytometry system (Becton-Dickinson, San Jose, CA) and the data were analyzed with BD FACSDiva Software v6.1.3.

### Statistical analyses

Statistical analyses of the experimental data were conducted with Prism 3.0 (GraphPad Software Inc., San Diego, CA, USA). All results are presented as the means ± SD from at least three independent experiments. P < 0.05 was considered statistically significant.

## Additional Information

**How to cite this article**: Cui, Y. *et al.* The miR-20-Rest-Wnt signaling axis regulates neural progenitor cell differentiation. *Sci. Rep.* **6**, 23300; doi: 10.1038/srep23300 (2016).

## Supplementary Material

Supplementary Information

## Figures and Tables

**Table 1:** Primers used to construct luciferase reporter plasmids of Rest.

Gene name	Primer sequence	Product size (bp)
Rest 3′UTR (miR-20 sense)	Forward/SpeI: 5′- TCACTAGTCTTTATATAAAGTTAGCACTTT -3′ REVERSE/SPHI: 5′- TAGCATGCCAAAGTGCCCTCATAGGA -3′	1097
Rest 3′UTR (mir-20 mutated putative binding region)	Forward/SpeI: 5′- TATAAAGTTATCATTCTAAGATT -3′ Reverse/SphI: 5′- AGAATGATAACTTTATATAAAGCAGGC -3′	1097

**Table 2:** Primers used in qRT-PCR.

Gene symbol	Primer sequence(5′-3′)	Product size (bp)
Nestin (Rattus Norvegicus)	Forward: AGAGAAGCGCTGGAACAGAG; Reverse: AGGTGTCTGCAACCGAGAGT	234
Tuj1 (Rattus Norvegicus)	Forward: AGCAGATGCTGGCCATTCAGAGTA; Reverse: TAAACTGCTCGGAGATGCGCTTGA	174
Sox2 (Rattus Norvegicus)	Forward: AAAGGAGAGAAGTTTGGAGCCCGA; Reverse: GGGCGAAGTGCAATTGGGATGAAA	113
Vimentin (Rattus Norvegicus)	Forward: AGGTGGATCAGCTCACCAATGACA; Reverse: TCAAGGTCAAGACGTGCCAGAGAA	184
Map2 (Rattus Norvegicus)	Forward: GCAGCGCCAATGGATTTCCATACA; Reverse: TCCGTTGATCCCGTTCTCTTTGGT	104
Gapdh (Rattus Norvegicus)	Forward: AAGGGCTCATGACCACAGTC; Reverse: GTGAGCTTCCCATTCAGCTC	169
Rest (Rattus Norvegicus)	Forward: CTCTCGAAAGCTGAACTGGC Reverse: GGCCTTCTCCTTCGCTATCT	169
