# Supplementary material for: The miR-20-Rest-Wnt signaling axis regulates neural progenitor cell differentiation
Source: Sci Rep. 2016 Mar 21;6:23300. doi: 10.1038/srep23300 (PMC4800422; doi:10.1038/srep23300)
Supplement: Supplementary Information [file srep23300-s1.doc]

# The miR-20-Rest-Wnt signaling axis regulates neural progenitor cell differentiation

Yi Cui^a,b*^, Jin Han^b*^, Zhifeng Xiao ^b*^, Tong Chen^c,d^, Bin Wang^b^, Bing Chen^b^, Sumei Liu^b^, Sufang Han^b^, Yongxiang Fang^e^, Jianshu Wei^b^, Xiujie Wang^d^, Xu Ma^a#^, and Jianwu Dai^b#^

^a^ Reproductive and Genetic Center of National Research Institute for Family Planning, Beijing 100081, China

^b^ State key Laboratory of Molecular Developmental Biology, Institute of Genetics and Developmental Biology, Chinese Academy of Sciences, Beijing 100190, China

^c^ University of Chinese Academy of Sciences, Beijing 100049, China

^d^ The State Key Laboratory of Plant Genomics, Institute of Genetics and Developmental Biology, Chinese Academy of Sciences, Beijing 100101, China

^e^ State Key Laboratory of Veterinary Etiological Biology, Key Laboratory of Veterinary Public Health of Ministry of Agriculture，Lanzhou Veterinary Research Institute, CAAS，Lanzhou 730046, China

Correspondence: Jianwu Dai, PhD., Professor, Institute of Genetics and Developmental Biology, Chinese Academy of Sciences, 3 Nanyitiao, Zhongguancun, Beijing 100190, China. 86-010-82614426 (phone/fax), E-mail: jwdai@genetics.ac.cn; or Dr Xu Ma, PhD., Professor, Reproductive and Genetic Center of the National Research Institute for Family Planning, Beijing 100081, China. E-mail: genetic88@126.com ^*^These authors contributed equally to this work.

#Dr Jianwu Dai, Institute of Genetics and Developmental Biology, Chinese Academy of Sciences, 3 Nanyitiao, Zhongguancun, Beijing 100190, China (E-mail: jwdai@genentics.ac.cn) or Dr Xu Ma, Reproductive and Genetic Center of the National Research Institute for Family Planning, Beijing 100081, China (E-mail: [genetic88@126.com](mailto:genetic88@126.com))

FigS1. Wnt signaling regulates the neural differentiation of NPCs. Immunostaining images and quantified data of Nestin (A), Sox2 (B), Vimentin (C), Tuj1(D) and MAP2 (E) positive cells in NPCs cultured in medium containing Wnt3a ( or Dkk1) or transfected with β-catenin siRNA for 96h. Quantitation and representative photomicrographs showed that Wnt siganling promotes cell differentiation in NPCs. The datas are shown as the means±SD. from 3 independent repetitions. *P<0.05 versus ctr , **P<0.01 versus ctr, *** P < 0.001 vs. ctr.
